# Isolated Iliac Artery Aneurysm: A Narrative Review of Natural History and Treatment in the Era of Iliac Branch Devices

**DOI:** 10.3400/avd.ra.26-00125

**Published:** 2026-09-01

**Authors:** Katsuyuki Hoshina

**Affiliations:** Department of Vascular Surgery, The University of Tokyo, Tokyo, Japan

**Keywords:** iliac artery aneurysm, natural history, endovascular repair, internal iliac artery, iliac branch device

## Abstract

Isolated iliac artery aneurysm—defined as aneurysmal dilatation of the common, internal, or external iliac artery without a concurrent abdominal aortic aneurysm requiring simultaneous repair—accounts for approximately 2%–7% of intra-abdominal aneurysms. This narrative review examines its natural history and the relative merits of open and endovascular treatment, with particular attention to internal iliac artery management, drawing on literature published since 2007. Two features deserve greater attention. First, much of what is cited as evidence on isolated disease derives from mixed aorto-iliac cohorts: across 7 such series, isolated aneurysms accounted for 120 of 897 patients (13.4%). Second, the frequently repeated claim that Japanese patients experience rupture at smaller diameters rests on comparisons between treated referral cohorts and Western imaging-surveillance cohorts, whose denominators are not equivalent. Reported growth rates range from 0.2 to 1.6 mm/year and accelerate with increasing diameter. Endovascular repair offers consistent perioperative advantages, whereas open repair may prove more durable when internal iliac artery sacrifice would otherwise be required. Although iliac branch devices are widely used in clinical practice, only 97 patients with truly isolated disease have been reported in dedicated series. The positions on treatment thresholds and techniques presented in this review are therefore identified as the author’s opinions.

## Introduction

Isolated iliac artery aneurysm (IAA) is defined as aneurysmal dilatation of the common, internal, and/or external iliac artery in the absence of a concurrent untreated abdominal aortic aneurysm (AAA) requiring simultaneous repair. It is uncommon, accounting for roughly 2%–7% of intra-abdominal aneurysms, and it is unusual among vascular conditions in that its low incidence has never been matched by a correspondingly modest confidence in how it should be managed. Diameter thresholds are quoted with precision, technique preferences are asserted with conviction, and both rest on a literature that is smaller and less specific than its frequent citation suggests.

Since the late 2000s, iliac branch devices, solitary iliac branch endoprostheses, and related stent-graft technologies have substantially expanded treatment options and changed the way the internal iliac artery (IIA) is approached. Whether that change has translated into better outcomes for patients with isolated disease, as opposed to patients with aorto-iliac disease in whom the same devices are deployed, is a question that the literature answers less clearly than is generally assumed.

Two systematic reviews have addressed adjacent questions—endovascular repair of isolated common iliac artery aneurysm (CIAA) with suggested diameter thresholds^1)^ and the surgical and endovascular management of isolated internal iliac artery aneurysm (IIAA)^2)^—while the 2024 European Society for Vascular Surgery guidelines cover iliac aneurysm within its aorto-iliac remit.^3)^

### Scope of this review

This is a narrative review. It draws on English-language reports published from January 2007 onward, corresponding to the period during which branch and stent-graft technology became materially relevant to treatment selection for this condition. Studies were identified through searches of PubMed and the Cochrane Library, supplemented by citation tracking from the reviews cited above and from the 2024 guideline. Reports involving fewer than 10 patients were not used.

It is not a systematic review, and it is not presented as one. No protocol was registered, selection was performed by a single author, no formal risk-of-bias assessment or certainty grading was undertaken, and no quantitative synthesis was attempted. The purpose is 3-fold: to describe the shape of the available literature; to draw attention to 2 features of that literature which, in the author’s view, are under-recognized and affect how the rest of it should be read; and to set out how the author approaches these patients in practice. Where a clinical position is taken, it is identified as the author’s opinion and should be weighed as such.

### How much of the evidence is actually about isolated disease?

It is worth establishing at the outset how much of the literature routinely cited for isolated IAA in fact concerns patients with isolated IAA.

Seven series report outcomes for aorto-iliac and isolated iliac aneurysms together without a separately reported isolated subgroup (**Table 1**).^4–10)^ Together, they enrolled 897 patients, of whom 120 met the isolated definition—13.4%, with the isolated proportion in individual series ranging from approximately 4% to 30%. Two of these are the largest iliac branch device series in existence: the pELVIS registry of 575 patients across 6 European centers with 8 years of follow-up, of whom 79 (13.7%) had isolated disease,^5)^ and a Dutch-led multicenter registry of 140 patients, of whom 17 (12.1%) had isolated disease.^4)^

**Table 1:** Series in which isolated aneurysms were a minority of the cohort

Study (ref.)	Country	Total N	Isolated n (%)	Comment
Jongsma et al.^4)^	Netherlands	140	17 (12.1)	Multicenter iliac branch device registry; outcomes pooled, not isolated-specific
Donas et al., pELVIS registry^5)^	6 European centers	575	79 (13.7)	Largest iliac branch device registry identified, with 8-yr follow-up; outcomes pooled
Huang et al.^6)^	Taiwan	46	2 (4.3)	Commercial vs. custom-made iliac branch devices; nearly all patients had AAA
Kliewer et al.^7)^	Austria	46	7 (15.2)	Outcomes reported by treatment method, not by isolated status
Walter et al.^8)^	Austria	54	7 (13.0)	Stent-graft occlusion risk-factor study; outcomes pooled
Machado et al.^9)^	Portugal	16	2 (12.5)	Small series; outcomes pooled
Raslan et al.^10)^	Egypt	20	6 (30.0)	Prospective series; 70% had concomitant AAA
Total		897	120 (13.4)	

These series are the source of most of the published device experience cited for isolated iliac artery aneurysm; approximately 6 of every 7 contributing patients did not have isolated disease.

AAA, abdominal aortic aneurysm

The consequence is structural rather than incidental. Because these registries dominate the published experience with iliac branch devices, and because they are commonly cited alongside genuinely isolated series, prevailing impressions of device performance in isolated IAA are formed largely from patients in whom the device was deployed as 1 component of an aorto-iliac repair. Those patients differ in the anatomy of the proximal landing zone, the presence of an aortic stent-graft body, the technical demands of the procedure, and the surveillance that follows. Approximately 6 of every 7 patients contributing to that experience did not have the condition under discussion.

By contrast, only 2 series have reported iliac branch device outcomes in a population that was entirely isolated: an Italian 7-center registry of 46 patients with 49 aneurysms,^11)^ and an eighteen-center international registry of 51 patients with 54 aneurysms.^12)^ These 97 patients do not represent the total clinical use of iliac branch devices; rather, they constitute the entirety of the published experience with IIA flow preservation in truly isolated disease reported in dedicated series.

This is not an argument that the mixed-cohort data are uninformative. They are the best technical evidence available for these devices and are properly cited as such. It is, however, an argument for reading the isolated-disease literature with an accurate sense of how thin it is and for resisting the temptation to treat registry-scale device experience as though it were registry-scale experience of this condition.

## Natural History

Six reports contribute natural-history or growth data (**Table 2**).^13–18)^

**Table 2:** Natural history and growth of isolated iliac artery aneurysm

Study (ref.)	Country	N	Key finding
Steenberge et al.^13)^	USA	167 pts/244 CIAA	Growth rates were 0.2, 0.3, and 1.3 mm/yr for 20–24.9, 25–29.9 and ≥30 mm, respectively (0.5 mm/yr at ≥30 mm excluding 2 outliers); no rupture or symptomatic presentation during a mean 62 mo surveillance; proposed threshold of 3.5 cm
Laine et al.^14)^	Multinational (VASCUNET)	66 (28% isolated)	Rupture was rare below 4 cm; proposed a 4-cm elective threshold for IIAA
Yoshino et al.^15)^	Japan	83	Quadratic (accelerating) growth with diameter: 3.28 vs. 1.43 mm/yr above vs. below 30 mm, respectively (p <0.0001); rupture occurred at 30.1 mm in a patient who declined surgery
Dhanji et al.^16)^	United Kingdom	215 CIAA (23% isolated)	Mean growth 1.5 mm/yr; growth above 30 mm was unpredictable
Hiromatsu et al.^17)^	Japan	41 pts/53 IAAs	20 of 41 patients (49%) presented with rupture over 21 yr in a treated referral cohort, vs. 8% for concurrent AAA at the same institution (p = 0.0008); 30-d mortality 9.8%, entirely in ruptured cases
Huang et al., isolated subgroup^18)^	USA	46 isolated of 438	Isolated-subgroup expansion was 0.40 cm/yr, vs. 0.27 cm/yr for the pooled cohort; this finding was based on 10 aneurysms with serial imaging

Note the difference in denominator between the surveillance cohorts (refs. 13, 16) and the treated referral cohorts (refs. 15, 17); the 2 do not measure the same quantity.

CIAA, common iliac artery aneurysm; IAA, iliac artery aneurysm; IIAA, internal iliac artery aneurysm; AAA, abdominal aortic aneurysm

### Growth

The largest surveillance series, from the Cleveland Clinic, followed 244 isolated CIAAs in 167 patients for a mean of 62 months and found growth to be slow and strongly diameter-dependent: 0.2 mm/year for aneurysms of 20–24.9 mm, 0.3 mm/year for those of 25–29.9 mm, and 1.3 mm/year for those of 30 mm or more—the last figure falling to 0.5 mm/year when 2 extreme outliers were excluded.^13)^ A United Kingdom duplex-surveillance audit reported a mean growth rate of 1.5 mm/year,^16)^ and a Japanese single-center cohort reported a rate of 1.59 mm/year overall.^15)^ The isolated subgroup in a Mayo Clinic series expanded at 0.40 cm/year, compared with 0.27 cm/year in the pooled cohort, although that estimate was based on only 10 isolated aneurysms with serial imaging.^18)^

The spread—roughly 8-fold between the lowest and highest figures—is largely explained by case mix rather than by disagreement. Yoshino and colleagues showed directly that growth accelerates with diameter rather than proceeding linearly, at 3.28 mm/year above 30 mm vs. 1.43 mm/year below that threshold.^15)^ A series weighted toward small aneurysms will therefore report a low mean, whereas a series weighted toward larger aneurysms will report a high mean; the 2 may appear to conflict when they do not. It follows that a single “growth rate” for isolated CIAA is not a useful quantity and that surveillance intervals should be set according to current diameter rather than an assumed rate. The Cleveland Clinic authors proposed triennial imaging below 25 mm, biennial imaging up to 30 mm, and annual imaging thereafter,^13)^ which seems to the author to be a reasonable schedule and is the one used in his own practice.

### Rupture and proposed thresholds

Two groups have proposed thresholds. The Cleveland Clinic series observed no rupture or symptomatic presentation among 244 aneurysms during surveillance and recommended elective repair at 3.5 cm, while noting that further data were needed to support that figure.^13)^ A VASCUNET analysis of IIAA found rupture to be rare below 4 cm and proposed 4 cm as the elective threshold for that location.^14)^

Both proposals derive from the absence of observed events below the proposed diameter, which is different from a diameter-stratified estimate of rupture risk. No study in this literature has reported the rate of rupture per patient-year at a defined diameter, in any population. The 3.0–4.0 cm range in common use is therefore best understood as a pragmatic consensus informed by the absence of contrary evidence and broadly consistent with contemporary guidance,^3)^ rather than as a figure derived from measured risk. The distinction matters when a patient asks how much safer surgery makes them, because the honest answer is that the magnitude of the risk being avoided has not been quantified.

### The apparent difference between Japanese and Western cohorts

Japanese series are frequently cited as demonstrating a higher rupture risk than Western ones, and this observation has been used to argue for a lower intervention threshold in Japanese patients. The comparison does not survive examination of the denominators.

The Japanese figure usually quoted is a rupture rate of 49%—20 of 41 patients—over 21 years at a single institution, significantly higher than the 8% recorded for concurrent AAA at the same center, with 30-d mortality of 9.8% confined entirely to ruptured cases.^17)^ That denominator is the set of patients referred to and treated at a tertiary vascular unit. The figure therefore describes the proportion of a treated cohort that presented with rupture; it is a case-mix statistic. The Western figure with which it is contrasted—most prominently, the complete absence of rupture among 244 aneurysms under imaging surveillance^13)^—has as its denominator the set of patients under observation and describes the incidence of rupture during surveillance. These are not the same measure. A referral-based cohort will report a higher proportion of ruptured cases than a surveillance-based cohort even when the underlying rupture risk is identical, because patients who never rupture and are never operated on enter neither the numerator nor the denominator of the former.

The second Japanese observation, a rupture at 30.1 mm in a patient who had declined elective surgery,^15)^ is a single event. Sub-threshold ruptures have been reported in Western series as well; Western surveillance cohorts have generally reported rupture only in aggregate, so such events are less visible there rather than necessarily less frequent.

The author’s position is therefore that the question of whether rupture risk at a given diameter differs between Japanese and Western patients has not been studied and that the appearance that it has been answered arises from the juxtaposition of 2 incompatible study designs. This matters beyond the specific case. Where the same clinical question is addressed by referral-based cohorts in 1 country and surveillance-based cohorts in another, apparent national differences in disease behavior should be attributed to study design until a common denominator has been established. Testing the proposition here would require a Japanese surveillance cohort assembled at the point of imaging diagnosis rather than at the point of referral for treatment—a study that could be undertaken within the existing Japanese registry infrastructure and that the author would regard as the single most useful contribution now available in this field.

## Open vs. Endovascular Repair

Five series have compared open surgical repair (OSR) with endovascular repair directly in isolated IAA populations (**Table 3**).^19–23)^ A United States administrative-database analysis of 33161 admissions provides population-level mortality data.^24)^

**Table 3:** Studies comparing open surgical and endovascular repair

Study (ref.)	Country	N	Key finding
Chaer et al.^19)^	USA	71 (19 OSR/52 endovascular)	Lower transfusion requirement and shorter LOS with endovascular repair; no difference in mortality or freedom from secondary intervention
Patel et al.^20)^	USA	56 (24 OSR/32 endovascular)	5-yr primary patency 100% with OSR vs. 96% with endovascular repair (NS); substantial LOS advantage for endovascular repair
Igari et al.^21)^	Japan	32 (20 OSR/12 endovascular)	Lower blood loss and shorter operative time with endovascular repair; ruptured cases were excluded
Zhorzel et al.^22)^	Germany	94 pts/106 IAAs	Freedom from reintervention at 5 yr was 97% with OSR vs. 40% with endovascular repair (p <0.01); less endoleak and less buttock claudication occurred after OSR
Choi and Kwon^23)^	South Korea	58 (25 OSR/33 endovascular)	Late complications were more frequent after endovascular treatment (15.2% vs. 0%, p = 0.04)

No study adjusted for aneurysm diameter, urgency of presentation, or internal iliac artery management, all of which differed between treatment groups.

OSR, open surgical repair; LOS, length of stay; NS, not significant; IAA, iliac artery aneurysm

Perioperative outcomes consistently favor endovascular treatment. Reported advantages include lower blood loss and shorter operative time,^21)^ shorter hospital stay,^19,20)^ and a lower transfusion requirement.^19)^ In-hospital mortality in the national dataset was 0.5% after endovascular repair and 1.8% after open repair.^24)^ Perioperative mortality did not differ significantly between approaches in most single-center reports, in which event numbers were small.

Durability findings are less consistent, and the inconsistency is instructive. Zhorzel and colleagues, in the largest single-center comparison, reported freedom from reintervention of 97% after open repair vs. 40% after endovascular repair at 5 years, together with less endoleak and less buttock claudication.^22)^ Choi and Kwon likewise found late complications to be more frequent after endovascular treatment, at 15.2% vs. 0%, and recommended open repair as a first-line option in younger, lower-risk patients.^23)^ Patel and colleagues, by contrast, found 5-year primary patency of 100% after open repair and 96% after endovascular repair, a nonsignificant difference,^20)^ and Chaer and colleagues found no difference in freedom from secondary intervention.^19)^

The 57-percentage-point difference reported by Zhorzel is substantially larger than that reported by any other comparative series and larger than the endovascular reintervention rates reported in the single-arm literature would predict. None of the comparative series adjusted for aneurysm diameter, urgency of presentation, or IIA management, all of which differed between treatment groups and independently influenced the outcomes measured. Nor did any study define the indication for reintervention or the surveillance intensity applied to each group, and a cohort followed more closely after endovascular repair will accumulate more detected reinterventions for that reason alone. The safest interpretation is that endovascular repair carries a real and reproducible perioperative advantage, that a durability disadvantage is plausible and supported by 2 of 5 series, and that its magnitude remains unknown.

## Management of the Internal Iliac Artery

Whether the IIA is preserved, embolized, or covered is, in the author’s view, the decision that most strongly influences long-term outcome in this condition, and it is the axis along which the open-vs.-endovascular comparison should truly be drawn.

### Flow preservation

Two series have evaluated iliac branch devices used without an accompanying aortic stent-graft component in truly isolated populations (**Table 4**). Giaquinta and colleagues, reporting an Italian 7-center registry of 46 patients, observed no buttock claudication, no erectile dysfunction, and no bowel or spinal cord ischemia, with 95.7% freedom from reintervention at both 1 and 5 years.^11)^ The SIBES Study Group, reporting on 51 patients from eighteen international centers, similarly observed no buttock claudication, colonic ischemia, or gluteal or perineal necrosis, with 98.1% IIA and external iliac artery patency at 24 months.^12)^

**Table 4:** Iliac branch devices in truly isolated populations

Study (ref.)	Country	N	Key finding
Giaquinta et al.^11)^	Italy (7 centers)	46 pts/49 CIAA	No buttock claudication, erectile dysfunction, or bowel or spinal cord ischemia; 95.7% freedom from reintervention at 1 and 5 yr
Oussoren et al., SIBES Study Group^12)^	18 international centers	51 pts/54 IAAs	No buttock claudication, colonic ischemia, or gluteal or perineal necrosis; 98.1% IIA and EIA patency at 24 mo

These 2 series, totaling 97 patients, do not represent the total clinical use of iliac branch devices; rather, they constitute the published experience with internal iliac artery flow preservation in truly isolated disease reported in dedicated series. Neither study included a comparator group.

CIAA, common iliac artery aneurysm; IAA, iliac artery aneurysm; IIA, internal iliac artery; EIA, external iliac artery

These results are as good as could be hoped for, and they are the reason flow preservation has become the preferred approach where anatomy permits. Two qualifications are worth stating plainly. The first is simple arithmetic: in the pooled published cohort of 97 patients with no events, the upper 95% confidence bound on the complication rate is about 3%, which is reassuring but not equivalent to zero. The second is that neither series included a comparator group, and patients anatomically suitable for a branch device differ systematically from those who are not—in landing-zone quality, in tortuosity, and often in age. No study has compared preservation with sacrifice in an isolated population, so the magnitude of the benefit attributable to the technique, as distinct from the patients selected for it, has not been established.

### Embolization and coverage

Six series address IIAA treated by embolization, stent-graft coverage, or both (**Table 5**).^25–30)^ Buttock claudication, the principal morbidity of IIA sacrifice, was reported in 15% of patients in a French series^26)^ and 15.6% in a Chinese series,^29)^ the 2 reports that stated it explicitly. No series in this literature applied a prespecified definition of buttock claudication or a systematic schedule of symptom enquiry, and ascertainment in retrospective work depends on the symptom being volunteered by the patient and recorded by the clinician. The figure of approximately 15%–16% should therefore be regarded as a lower bound rather than a true estimate.

**Table 5:** Embolization and internal iliac artery coverage

Study (ref.)	Country	N	Key finding
Muradi et al.^25)^	Japan	33 pts/35 IIAA	Procedural success 97.1%; no aneurysm-related mortality
Pirvu et al.^26)^	France	20	No perioperative deaths; buttock claudication 15%
Millon et al.^27)^	France (multicenter)	53 pts/57 IIAA	No IIAA rupture during follow-up; cumulative survival 59% at 5 yr
Yang et al.^28)^	China	42 pts/43 IIAA	Combined embolization plus stent graft gave the best outcomes of 4 methods compared
Liu et al.^29)^	China	45 pts/49 IIAA	Reintervention-free survival 95.5% and 88.2% at 12 and 24 mo; buttock claudication 15.6%
Tsilimparis et al.^30)^	Germany	11 pts/12 IIAA	100% technical and clinical success; 2 early limb thromboses

No study stated a prespecified definition of buttock claudication or used a systematic schedule of symptom enquiry.

IIAA, internal iliac artery aneurysm

Perioperative mortality in this group was generally low,^25,26,28)^ and no IIAA-specific rupture occurred during follow-up in the largest embolization series, which comprised 53 patients across French centers.^27)^ Yang and colleagues, comparing 4 techniques, found combined embolization with stent-graft coverage to yield the best results among those examined, suggesting that technical refinement may mitigate this morbidity without abolishing it.^28)^

## Ruptured Presentation

Two reports address rupture outcomes specifically.^17,31)^ In a series of 26 open repairs (**Table 6**), early postoperative mortality was 6.7% among the 15 patients without rupture and 0% among the 11 patients with rupture, although the ruptured group had significantly higher inflammatory markers, greater blood loss, and higher transfusion requirements.^31)^ Both 30-d deaths in the German comparative cohort occurred in patients with rupture,^22)^ and the Japanese series discussed above reported 30-d mortality of 9.8% confined entirely to ruptured cases.^17)^

**Table 6:** Additional endovascular series, rupture-specific and population-level data

Study (ref.)	Country	N	Key finding
Chemelli et al.^32)^	Austria	91 pts/109 IAAs	Largest single-center series; primary technical success 90.1%; survival 47.6% at 10 yr
Kim et al.^33)^	South Korea	31	Technical success 100%; type II endoleak 16.1%; no major complications
Fossaceca et al.^34)^	Italy	45 pts/59 aneurysms	Technical success 97.8%; primary patency 95.5%; perioperative mortality 4.7%
Wang et al.^35)^	China	26 pts/72 aneurysms	100% technical success; combined CIAA and IIAA endovascular series
Stroumpouli et al.^36)^	United Kingdom	21	100% technical success; 0% 30-d mortality; 100% patency at 51.2 mo
Kim et al.^37)^	South Korea	49	Progressive aortic enlargement after straight (non-branched) stent grafts—long-term safety signal
Buck et al.^24)^	USA (National Inpatient Sample)	33161	In-hospital mortality 0.5% endovascular vs. 1.8% open (p <0.001)
Yamamoto et al.^31)^	Japan	26 (11 ruptured)	Early postoperative mortality 0% in ruptured vs. 6.7% in unruptured patients; ruptured group had higher inflammatory markers, greater blood loss and higher transfusion requirement
Hu et al.^38)^	Japan	28 pts/42 aneurysms	5- and 10-yr survival 90.5% and 75.4%, better than the concurrent AAA comparator

CIAA, common iliac artery aneurysm; IAA, iliac artery aneurysm; IIAA, internal iliac artery aneurysm; AAA, abdominal aortic aneurysm

These findings do not agree, and the disagreement should be reported rather than resolved. The 3 reports differ in era, in the proportion of patients presenting with hemodynamic instability, and—critically and without clear reporting—in whether patients who died before reaching the operating theatre were counted. Rupture may well remain an important determinant of mortality in this condition, and the author believes that it does, but the available series do not permit its magnitude to be estimated. No dedicated series of endovascular repair for ruptured isolated IAAs meeting the 10-patient threshold was identified, which is itself a notable gap given that endovascular repair is now the default approach to ruptured aneurysm in many centers.

## Additional Endovascular Series

Several further single-center and multicenter endovascular series, although not comparative, broaden the picture (**Table 6**).^32–38)^ Technical success was generally high and perioperative mortality was low. The largest single-center experience, from Austria, comprised 109 aneurysms in 91 patients, with 90.1% primary technical success and 47.6% survival at 10 years—a reminder that these patients die from the comorbidities that accompany aneurysmal disease rather than from the aneurysm that has been treated.^32)^

One South Korean report raises a long-term safety consideration that has attracted less attention than it merits: progressive proximal aortic enlargement after treatment with straight, non-branched stent grafts.^37)^ If confirmed, this finding has implications for device selection in younger patients and for the duration and modality of imaging follow-up, and it argues against regarding endovascular treatment of isolated iliac disease as a definitively completed intervention.

## The Author’s Approach

What follows is an opinion, offered because a review of this kind is of limited use if it declines to state what the author actually does. It is informed by the literature described above but is not derived from it in any formal sense, and readers should weigh it accordingly.

I place the threshold for elective repair of isolated CIAA at 35 mm in patients who are fit for intervention, and I do not lower it for Japanese patients, because I do not believe that evidence for a population-specific threshold exists. Below 35 mm, I follow the diameter-dependent surveillance schedule described above, with the caveat that I image annually rather than triennially in patients whose aneurysm has grown measurably between 2 prior studies, since accelerating growth is the 1 signal that has been demonstrated rather than assumed. For IIAA, I use a threshold of 35–40 mm, closer to 30 mm when the aneurysm is saccular or the patient is symptomatic.

Where anatomy permits IIA flow preservation, I preserve it. This is the position best supported by the evidence reviewed here, although it should be acknowledged that the support is uncontrolled. Where preservation is not feasible and unilateral sacrifice is required, I proceed and counsel the patient that roughly 1 in 6 will experience buttock claudication and that the true figure may be higher. I avoid bilateral sacrifice whenever any alternative exists.

I continue to offer open repair to younger patients with favorable anatomy and low operative risk, particularly when branch preservation would be difficult by endovascular means, and I explain the trade-off explicitly: a larger operation and a longer recovery vs. a reasonable expectation of not requiring another intervention. In patients over 75, or those with significant cardiopulmonary comorbidity, or those presenting with rupture, I treat endovascularly.

## Limitations of this Review

The limitations follow from the design and should be stated directly. This is a narrative review conducted by a single author. Study selection reflects that author’s judgment and was not duplicated; no protocol was registered; no formal appraisal of study quality was undertaken; and no attempt was made to weight or pool findings. Selection bias in what has been included and emphasized cannot be excluded, and a different author reviewing the same field would likely construct a somewhat different account.

The restriction to English-language publications is a particular limitation given the attention paid here to Japanese data, because Japanese-language series indexed in Ichushi-Web were not searched. The observations offered about the Japanese literature therefore concern the Japanese literature as it appears in English, which represents only a subset of it.

All of the underlying evidence is observational; no randomized trial addressing any question in this field has been reported, which is unsurprising given the rarity of the condition. Definitions of “isolated” differ between studies, with some excluding any history of AAA and others admitting secondary or anastomotic aneurysms after prior AAA repair, which limits comparison. Where this review states a clinical position, that position is the author’s own and carries the standing of experienced opinion rather than graded evidence.

## Conclusion

The literature on isolated IAA is smaller and less specific to the condition than its frequent citation implies, and 2 features of it deserve wider recognition. Most of the device experience routinely invoked for this condition comes from patients who did not have it, at a ratio of about 6 to 1. In addition, the reported difference in rupture risk between Japanese and Western patients is explicable by the difference between referral-based and surveillance-based study designs and has not, in fact, been tested.

Within those constraints, the reasonable positions are as follows. Growth is slow and diameter-dependent, so surveillance intervals should follow current diameter. Thresholds in the 3.0–4.0 cm range represent pragmatic consensus rather than measured risk. Endovascular repair carries a real perioperative advantage and a plausible but unquantified durability disadvantage. IIA flow preservation, where anatomy permits, is supported by high-quality but uncontrolled data of limited volume, and it is the author’s preferred approach. Where sacrifice is unavoidable, approximately 1 patient in 6 will experience buttock claudication, and the true figure is probably higher.

What would most improve this picture is not another single-center series. It is a surveillance cohort assembled at diagnosis rather than at referral, reporting rupture per patient-year at defined diameters, and a comparison of IIA preservation against sacrifice in patients with isolated disease.
